# Oral Candida Colonization Among Patients With Head and Neck Cancer Undergoing Radiotherapy and Its Impact on the Severity of Oral Mucositis: A Prospective Observational Study

**DOI:** 10.7759/cureus.110599

**Published:** 2026-06-10

**Authors:** Gutthi Rishitha Reddy, Sagayaraj A, Manjunath GN, SM Azeem Mohiyuddin, Arvind Natarajan

**Affiliations:** 1 Department of Otorhinolaryngology, Sri Devaraj Urs Medical College (SDUMC), Tamaka, Kolar, IND; 2 Department of Otorhinolaryngology and Head and Neck Surgery, Sri Devaraj Urs Medical College (SDUMC), Tamaka, Kolar, IND; 3 Department of Radiation Oncology, Sri Devaraj Urs Academy of Higher Education and Research, Sri Devaraj Urs Medical College (SDUMC), Tamaka, Kolar, IND; 4 Department of Microbiology, Sri Devaraj Urs Academy of Higher Education and Research, Sri Devaraj Urs Medical College (SDUMC), Tamaka, Kolar, IND

**Keywords:** candida colonization, head and neck cancer, oral candidiasis, oral mucositis, radiotherapy

## Abstract

Objective

Oral mucositis is a common complication of radiotherapy (RT) in patients with head and neck cancer (HNC), often resulting in reduced treatment tolerance and impaired quality of life. Radiation-induced mucosal injury predisposes patients to *Candida* colonization, which may influence the early onset and severity of mucositis. This study aimed to determine the prevalence and temporal pattern of oral *Candida* colonization in patients with head and neck cancer undergoing radiotherapy and to evaluate its association with the severity of radiation-induced oral mucositis while accounting for relevant clinical factors, including age, comorbidities, and treatment-related variables.

Methodology

A prospective observational study was conducted on 70 patients aged 40-80 years undergoing curative radiotherapy for head and neck cancers at a tertiary care facility. Sterile swabs were collected from multiple oral sites before radiotherapy, during the third week of treatment, and at the end of radiotherapy. Samples were inoculated on Sabouraud dextrose agar to identify *Candida* species. Oral mucositis was assessed weekly using the World Health Organization (WHO) and Common Terminology Criteria for Adverse Events (CTCAE) scales. Statistical analysis was performed using SPSS version 25 (IBM Corp., Armonk, NY), with associations evaluated using the chi-square or Fisher’s exact test.

Results

Among the 70 patients, 32 (45.7%) were *Candida-*positive by the third week of radiotherapy, progressing to 44 (62.9%) after treatment completion. Oral mucositis developed in 58 (82.9%) patients, with WHO grades 2 and 3 being the most frequent, affecting 22 (31.4%) and 16 (22.9%) patients, respectively. *Candida* positivity correlated with mucositis severity, observed in 14 (63.6%) patients with grade 2 and 14 (87.5%) patients with grade 3 mucositis. The oral cavity was the most common site of *Candida* colonization, affecting 20 (66.7%) patients. No patients were *Candida-*positive before radiotherapy.

Conclusion

Oral *Candida* colonization increased significantly during radiotherapy and was closely associated with the severity of oral mucositis. The early detection and management of fungal colonization may improve treatment tolerance, reduce mucosal injury, and enhance the quality of life in patients undergoing head and neck radiotherapy.

## Introduction

*Candida albicans* and other opportunistic oral fungi are major causes of oral candidiasis, particularly in immunocompromised individuals such as patients receiving radiotherapy (RT) for head and neck cancer (HNC) [1]. Radiotherapy is a cornerstone in the management of head and neck cancer, used as definitive or adjuvant treatment. However, it is associated with several acute and chronic oral complications that impair the quality of life, nutritional status, and treatment adherence. Among these, oral mucositis is one of the most debilitating toxicities [2].

Radiation-induced oral mucositis presents with erythema, ulceration, severe pain, and dysphagia. It also increases the risk of secondary infections. Its pathogenesis is multifactorial. Radiation causes epithelial injury, generates reactive oxygen species, and triggers the release of pro-inflammatory cytokines. These processes disrupt mucosal integrity, leading to painful ulcerations. The loss of barrier function facilitates microbial colonization, particularly by *Candida* species, which may further delay healing [3]. Radiotherapy also triggers hyposalivation, changes in oral pH, and the disruption of the normal oral microflora and contributes to nutritional deficiencies. All these factors predispose patients to fungal overgrowth and chronic *Candida* colonization [4]. Several studies have documented that the rates of oral *Candida* carriage increase significantly during and after radiotherapy, with prevalence rates of 40%-80% in patients with HNC [5-8]. Although colonization may be asymptomatic initially, it may later progress to clinically overt oral *candidiasis*, manifesting as pseudomembranous or erythematous lesions and angular cheilitis, thereby increasing patient morbidity [6].

Emerging evidence suggests a bidirectional relationship between the severity of oral mucositis and oral *Candida* colonization. A breakdown of mucosal barriers promotes fungal adhesion and invasion, whereas *Candida* infection promotes inflammation, epithelial damage, and pain, which may increase the severity of mucositis [7]. Adhesins, hydrolytic enzymes, and biofilm formation are *Candida* virulence factors that facilitate the long-term colonization of irradiated tissues and can disrupt epithelial regeneration [8,9]. Severe mucositis may lead to treatment interruptions, dose adjustments, and increased healthcare utilization, thereby undermining oncologic outcomes. Nonetheless, the precise influence of oral *Candida* colonization on the development and severity of radiation-induced mucositis remains unclear, especially in prospective studies [10]. Therefore, the present study was designed to evaluate the prevalence and progression of oral *Candida *colonization in patients with head and neck cancer undergoing radiotherapy and to assess its association with the severity of oral mucositis while accounting for relevant clinical and treatment-related factors. In this study, *Candida *colonization refers to the presence of fungal organisms without overt clinical infection, thereby distinguishing it from oral candidiasis.

## Materials and methods

Study design and setting

This prospective observational study was conducted in the Departments of Otorhinolaryngology and Head and Neck Surgery, Radiation Oncology, and Microbiology at RL Jalappa Hospital from January 2025 to January 2026.

Inclusion and exclusion criteria

Patients diagnosed with head and neck cancer and scheduled for curative radiotherapy were screened for eligibility. Patients aged 40-80 years with histopathologically confirmed head and neck cancer who were planned for definitive or adjuvant radiotherapy and provided written informed consent were included. Patients receiving palliative radiotherapy, those with a preexisting diagnosis of oral candidiasis, and those unable to undergo intraoral examination were excluded.

A consecutive sampling method was used, and all eligible patients presenting during the study period were enrolled until the required sample size was achieved.

Sample size calculation

The sample size was calculated based on the proportion of patients undergoing radiotherapy for head and neck cancers who develop oral mucositis, reported by Rupe et al. as 23.7% [11]. Assuming a 5% alpha error (95% confidence interval) and an absolute precision of 10%, the minimum required sample size to estimate the proportion of patients with oral mucositis was 70 subjects. This was determined using the standard formula for estimating proportions, where Z represents the critical value for a 95% confidence interval, P is the expected proportion, and d is the absolute precision. Accordingly, a minimum of 70 participants was enrolled in the study.

Study variables

The primary outcomes were the presence and frequency of oral *Candida* colonization and the severity of oral mucositis. In this study, *Candida* colonization was defined as the microbiological isolation of *Candida* species from oral swab samples in the absence of overt clinical signs of infection. Mucositis severity was assessed using the World Health Organization (WHO) grading system [12] and the Common Terminology Criteria for Adverse Events (CTCAE) [13]. Secondary variables included age, sex, tumor site, tumor stage, radiotherapy dose, and treatment duration. They selected clinical factors, including diabetes mellitus, anemia, and xerostomia, that may influence fungal colonization and mucosal toxicity.

Study procedure

Ethical approval was obtained from the Central Ethics Committee of Sri Devaraj Urs Academy of Higher Education and Research (approval number: SDUAHER/R&D/CEC/SDUMC-PG/19/NF/-2025-2026), and written informed consent was obtained from all participants before enrollment. Baseline oral examinations were performed before the start of radiotherapy, and participants were instructed to refrain from eating, drinking, smoking, or using oral hygiene products for at least one hour before sample collection.

Sterile swabs were collected from multiple oral sites, including the hard palate, tongue, upper and lower vestibules, and oral commissures. Samples were collected before radiotherapy, during the third week of treatment, and after the completion of radiotherapy. Each swab was placed in transport media and sent to the microbiology laboratory for analysis.

Microbiological Methods

Specimens were cultured on Sabouraud dextrose agar and incubated at 25°C-30°C for five to seven days or until fungal growth was observed.

In addition, incubation at 35°C-37°C was performed where required to optimize the recovery of clinically relevant *Candida* species.

*Candida* species were identified using standard microbiological techniques, including colony morphology, Gram staining, and germ tube testing. Chromogenic media and biochemical identification methods further supported applicable species differentiation in accordance with standard laboratory protocols.

Radiotherapy and Clinical Management

Radiotherapy was delivered using either a linear accelerator or a telecobalt machine, depending on machine availability and treatment planning considerations.

Patients received conventionally fractionated radiotherapy with a daily dose of 1.8-2 Gy, administered five days per week over six to seven weeks, to a cumulative dose of 60-70 Gy, depending on tumor site, stage, and treatment intent.

Detailed subgroup analysis based on radiotherapy technique (linear accelerator versus telecobalt) and concurrent chemotherapy was not within the scope of the present study.

All patients received standard supportive care before and during radiotherapy. Oral hygiene counseling was provided before treatment initiation, including instructions on gentle toothbrushing, saline or sodium bicarbonate mouth rinses, and the avoidance of local irritants such as tobacco and alcohol.

Mucositis was managed symptomatically using standard institutional protocols, including analgesics, topical agents, and supportive care measures. Patients who developed clinical features suggestive of oral candidiasis during radiotherapy received appropriate topical or systemic antifungal therapy according to institutional treatment protocols. However, routine antifungal prophylaxis was not administered universally to all patients during radiotherapy.

Preventive measures focused on maintaining oral hygiene, the early identification of mucosal changes, and managing xerostomia. Weekly oral examinations were conducted during treatment to document erythema, ulceration, pain, and healing status. Patients were followed for 4-12 weeks after radiotherapy to assess mucositis resolution and late effects.

Data collection

Data were recorded on a structured form that included demographic details, clinical findings, radiotherapy parameters, microbiological results, and mucositis grading. Oral mucositis was assessed weekly using the WHO [12] and CTCAE grading [13] systems. Mucositis grading was performed by trained clinicians using standardized WHO and CTCAE criteria. However, formal interobserver calibration analysis was not performed. Microbiological findings were documented at each sampling interval to evaluate temporal changes in fungal colonization.

Statistical analysis

Data were entered into Microsoft Excel (Microsoft Corp., Redmond, WA) and analyzed using SPSS version 25 (IBM Corp., Armonk, NY). Categorical variables were expressed as frequencies and percentages, while continuous variables were summarized as mean ± standard deviation or median, as appropriate. Normality of distribution was assessed using the Kolmogorov-Smirnov test. Nonparametric tests were applied for non-normally distributed data. Associations between *Candida* colonization and mucositis severity were analyzed using the chi-square test or Fisher’s exact test. A p-value of ≤0.05 was considered statistically significant.

## Results

Among 70 patients, 24 (34.3%) belonged to the 50-59-year age group, followed by 22 (31.4%) patients in the 60-69-year age group. Patients aged 40-49 years and 70-80 years each accounted for 12 (17.1%) cases. Male patients predominated, with 52 (74.3%) cases, whereas women comprised 18 (25.7%) cases. Regarding the site of tumor, 30 (42.9%) patients had tumors in the oral cavity, followed by 18 (25.7%) in the larynx, 13 (18.6%) in the oropharynx, and nine (12.9%) in the hypopharynx (Table 1).

**Table 1:** Sociodemographic characteristics and tumor site distribution of the study population (N = 70)

Category	Total number, N (%)
Age (years)	
40-49	12 (17.1%)
50-59	24 (34.3%)
60-69	22 (31.4%)
70-80	12 (17.1%)
Sex	
Male	52 (74.3%)
Female	18 (25.7%)
Site of tumor	
Oral cavity	30 (42.9%)
Oropharynx	13 (18.6%)
Hypopharynx	9 (12.9%)
Larynx	18 (25.7%)
Total	70 (100%)

Among the studied factors, age greater than 60 years was significantly associated with *Candida* positivity, as 28 (63.6%) patients were *Candida-*positive (χ² = 4.16; p = 0.041). Anemia also showed a significant association, with 26 (59.1%) *Candida*-positive patients (χ² = 4.58; p = 0.032). Xerostomia demonstrated the strongest association with *Candida* positivity, as 34 (89.5%) patients with xerostomia were *Candida-*positive (χ² = 18.72; p < 0.001). However, diabetes mellitus and hypertension did not show a statistically significant association with *Candida* positivity (p > 0.05) (Table 2).

**Table 2:** Contributing factors associated with oral Candida colonization *Statistically significant association (p < 0.05)

Factor	Number of patients	*Candida-*positive (n = 44)	Chi-square value (χ²)	P-value
Age (>60years)	34	28 (63.6%)	4.16	0.041*
Diabetes mellitus	28	18 (40.9%)	1.78	0.182
Hypertension	25	18 (40.95%)	1.25	0.264
Anemia	30	26 (59.1%)	4.58	0.032*
Xerostomia	38	34 (89.5%)	18.72	<0.001*

Among patients receiving radiotherapy, 30 underwent definitive RT, and 40 received adjuvant RT. In the definitive RT group, eight patients had oral cavity tumors, five had oropharyngeal tumors, five had hypopharyngeal tumors, and 12 had laryngeal tumors. In the adjuvant RT group, 22 patients had oral cavity tumors, eight had oropharyngeal tumors, four had hypopharyngeal tumors, and six had laryngeal tumors (Table 3).

**Table 3:** Subsite-wise distribution of definitive and adjuvant radiotherapy RT: radiotherapy

Site	Definitive RT (n = 30)	Adjuvant RT (n = 40)
Oral cavity	8	22
Oropharynx	5	8
Hypopharynx	5	4
Larynx	12	6
Total	30	40

At the third week of radiotherapy, *Candida* positivity was observed in 16 out of 30 patients with oral cavity tumors, six out of 13 patients with oropharyngeal tumors, four out of nine patients with hypopharyngeal tumors, and six out of 18 patients with laryngeal tumors. Overall, 32 patients were *Candida-*positive, and 38 were *Candida-*negative. However, no statistically significant association was observed between tumor site and *Candida* positivity at the third week of radiotherapy (χ² = 3.82; p = 0.28). At the completion of radiotherapy, *Candida* positivity increased to 20 out of 30 patients with oral cavity tumors, nine out of 13 patients with oropharyngeal tumors, five out of nine patients with hypopharyngeal tumors, and 10 out of 18 patients with laryngeal tumors. Overall, 44 patients were *Candida-*positive, and 26 were *Candida-*negative. Nevertheless, the association between tumor site and *Candida* positivity remained statistically nonsignificant (χ² = 4.67; p = 0.19) (Table 4).

**Table 4:** Candida positivity according to site at the third week and at the completion of radiotherapy

Site	Total patients	*Candida*-positive	*Candida*-negative	Chi-square value (χ²)	P-value
At third week of radiotherapy				3.82	0.28
Oral cavity	30	16	14		
Oropharynx	13	6	7		
Hypopharynx	9	4	5		
Larynx	18	6	12		
Total	70	32	38		
At completion of radiotherapy				4.67	0.19
Oral cavity	30	20	10		
Oropharynx	13	9	4		
Hypopharynx	9	5	4		
Larynx	18	10	8		

No *Candida* positivity was observed before radiotherapy, as all 70 (100%) patients were *Candida-*negative. At the third week of radiotherapy, 32 (45.7%) patients became *Candida-*positive, while 38 (54.3%) remained *Candida-*negative. Following the completion of radiotherapy, *Candida* positivity further increased to 44 (62.9%) patients, whereas 26 (37.1%) patients were *Candida-*negative. The increase in *Candida* positivity across different time points was found to be statistically significant (χ² = 31.84; p < 0.001) (Table 5).

**Table 5:** Candida colonization at different time points during radiotherapy (N = 70) *Statistically significant association (p < 0.05)

Time point	*Candida*-positive, n (%)	*Candida*-negative, n (%)	Chi-square value (χ²)	P-value
Pre-radiotherapy	0 (0.0)	70 (100%)	31.84	<0.001*
Third week of radiotherapy	32 (45.7)	38 (54.3)		
Post-radiotherapy	44 (62.9)	26 (37.1)		

Oral mucositis developed in 58/70 (82.9%) patients during radiotherapy. At the third week of treatment, WHO grade 2 mucositis was observed in 22/70 (31.4%) patients, while grade ≥3 mucositis was observed in 22/70 (31.4%) patients. At the completion of radiotherapy, CTCAE grade 2 mucositis was present in 24/70 (34.3%) patients, whereas CTCAE grade ≥3 mucositis was observed in 26/70 (37.1%) patients (Table 6).

**Table 6:** Distribution of mucositis status and severity grades during and after radiotherapy (RT) WHO, World Health Organization; CTCAE, Common Terminology Criteria for Adverse Events

Mucositis status	Total number (N)	Frequency (%)
Present	58	82.9
Absent	12	17.1
WHO mucositis grades (week 3 of RT)		
Grade 0	8	11.4
Grade 1	18	25.7
Grade 2	22	31.4
Grade 3	16	22.9
Grade 4	6	8.6
CTCAE mucositis grades (post-RT)		
Grade 0	6	8.6
Grade 1	14	20.0
Grade 2	24	34.3
Grade 3	20	28.6
Grade 4	6	8.6

At the third week of radiotherapy, *Candida* colonization increased progressively with higher WHO mucositis grades. Among 18 patients with grade 1 mucositis, six (33.3%) were *Candida-*positive. Among 22 patients with grade 2 mucositis, 14 (63.6%) were *Candida-*positive. Of the 16 patients with grade 3 mucositis, 14 (87.5%) demonstrated *Candida* colonization. Notably, all six (100%) patients with grade 4 mucositis were *Candida-*positive, indicating a clear positive correlation between increasing mucositis severity and fungal colonization (Table 7).

**Table 7:** Candida positivity according to WHO mucositis grade during the third week of radiotherapy WHO: World Health Organization

WHO grade	Total patients	*Candida*-positive, n (%)
Grade 1	18	6 (33.3%)
Grade 2	22	14 (63.6%)
Grade 3	16	14 (87.5%)
Grade 4	6	6 (100%)

Among the 44 *Candida*-positive patients, 26 (59.1%) developed severe mucositis, while 18 (40.9%) had mild or no mucositis. In contrast, among the 26 *Candida*-negative patients, only six (23.1%) developed severe mucositis, whereas 20 (76.9%) had mild or no mucositis. Overall, severe mucositis was observed in 32 of 70 patients, while 38 of 70 had mild or no mucositis. The association between *Candida* positivity and severe mucositis was statistically significant (χ² = 8.12; p = 0.004) (Table 8).

**Table 8:** Association between Candida colonization and severe mucositis (WHO grade ≥3) A p-value of less than 0.05 was considered significant *Significant p-value WHO: World Health Organization

*Candida* status	Severe mucositis, n (%)	Mild/no mucositis, n (%)	Total	Chi-square	P-value
Positive	26 (59.1)	18 (40.9)	44	8.12	0.004*
Negative	6 (23.1)	20 (76.9)	26		
Total	32	38	70		

**Figure 1:**
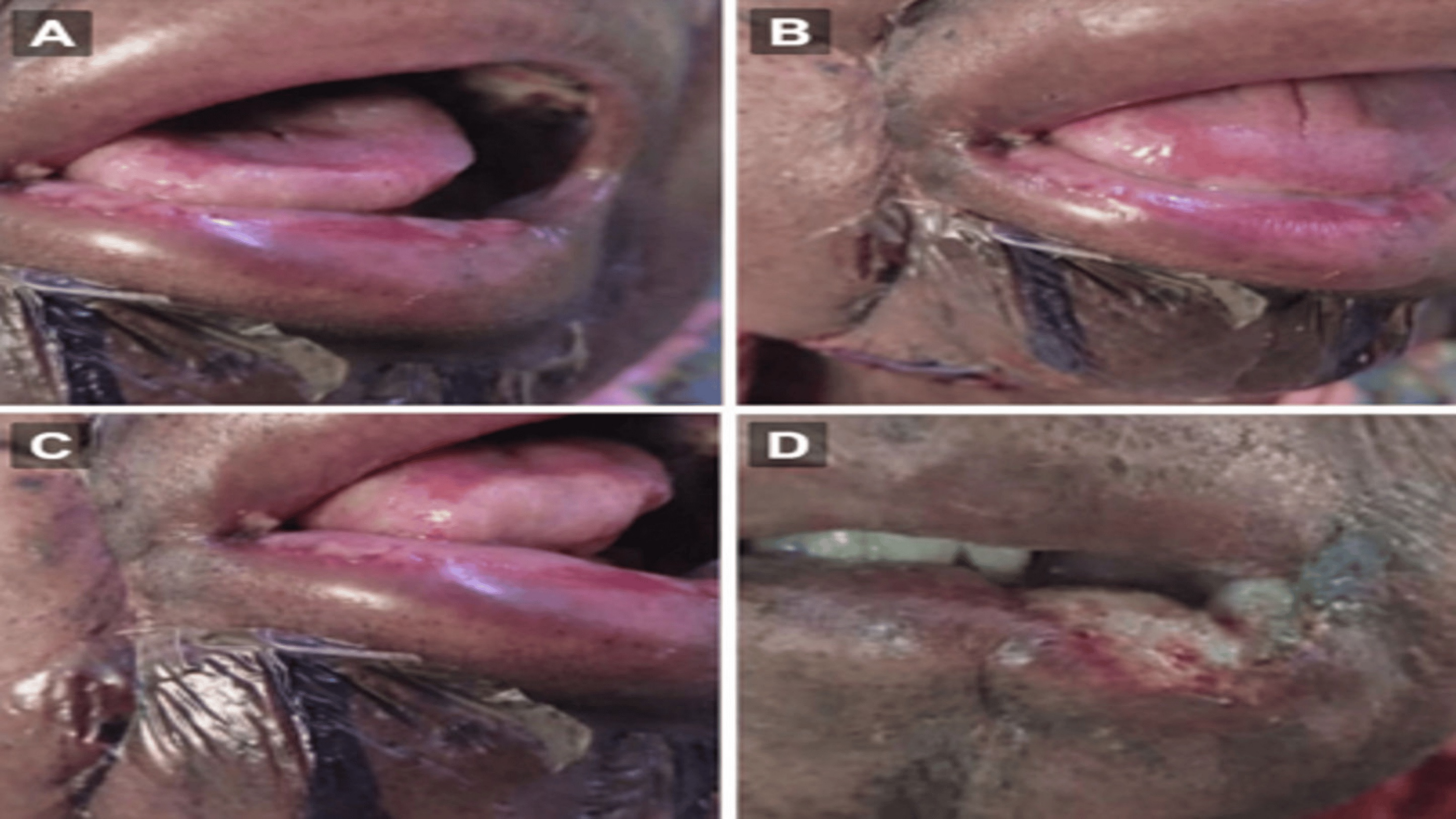
Clinical presentation of grade 3 radiation-induced oral mucositis in patients with head and neck cancer (A) Extensive erythema and ulceration involving the lower lip and adjacent buccal mucosa. (B) Diffuse mucosal erythema with epithelial sloughing affecting the oral commissure and inner lip. (C) Marked inflammation with ulcerative lesions involving the labial mucosa and perioral region. (D) Confluent ulceration with mucosal breakdown and erythematous base consistent with severe mucosal injury

Figure 1 demonstrates composite clinical photographs of grade 3 oral mucositis in patients undergoing radiotherapy for head and neck cancer. The images reveal extensive mucosal erythema, confluent ulcerations, and epithelial breakdown involving the lips, buccal mucosa, and oral commissures, consistent with severe mucosal toxicity requiring active symptomatic management.

Before the initiation of radiotherapy, zero (0%) of 70 patients were *Candida*-positive with grade ≥3 mucositis. At the third week of radiotherapy, 14 (20%) of 70 patients were *Candida-*positive with grade ≥3 mucositis. After radiotherapy, 26 (37.1%) of 70 patients were *Candida*-positive with grade ≥3 mucositis (Table 9).

**Table 9:** Temporal relationship between Candida colonization and mucositis severity RT: radiotherapy

Time point	*Candida*-positive with grade ≥3 mucositis, n (%)
Pre-RT	0 (0.0)
Week 3 of RT	14 (20.0)
Post-RT	26 (37.1)

At 12 weeks post-radiotherapy, 14 (20.0%) of 70 patients had persistent candidiasis, 30 (42.9%) had resolved candidiasis, and 26 (37.1%) had never developed candidiasis (Table 10).

**Table 10:** Persistence of oral candidiasis at 12 weeks post-radiotherapy

Candidiasis status at 12 weeks	Number (n)	Percentage (%)
Persistent candidiasis	14	20.0%
Resolved candidiasis	30	42.9%
Never developed candidiasis	26	37.1%
Total	70	100%

## Discussion

Radiation-induced oral mucositis is one of the most frequent and clinically significant complications of radiotherapy in patients with head and neck cancer [8]. Identifying modifiable factors that influence its severity is essential to improve patient comfort, treatment compliance, and overall outcomes. The present study demonstrates a statistically significant association between oral *Candida* colonization and the severity of radiation-induced oral mucositis, suggesting that fungal overgrowth may exacerbate mucosal injury during radiotherapy.

Our findings demonstrated that none of the patients showed detectable oral *Candida* colonization before the initiation of radiotherapy. Although baseline oral *Candida* carriage has been reported in healthy individuals and patients with cancer in previous studies, the absence of detectable colonization in the present cohort may be attributable to multiple factors, including pre-radiotherapy oral hygiene counseling, the exclusion of patients with overt oral candidiasis, the possible prior use of oral hygiene measures or antimicrobial agents, and the use of culture-based microbiological methods, which may have lower sensitivity for detecting low-level asymptomatic colonization. Variations in oral hygiene practices, dietary habits, sampling methodology, and regional population characteristics may also have contributed to the absence of detectable baseline colonization in this cohort. During radiotherapy, oral *Candida* colonization progressively increased, reaching 32/70 (45.7%) patients by the third week and 44/70 (62.9%) patients after the completion of radiotherapy, paralleling the progression of oral mucositis.

Radiotherapy remains a cornerstone in the management of head and neck cancers; however, its cytotoxic effects extend to normal tissues within the irradiation field. Ionizing radiation induces epithelial injury, generates reactive oxygen species, triggers the release of inflammatory cytokines, and disrupts mucosal integrity, ultimately culminating in oral mucositis. In this study, oral mucositis developed in 82.9% of patients, with 62.9% experiencing moderate-to-severe mucositis by the third week and 71.5% demonstrating CTCAE grade ≥2 mucositis posttreatment. Severe mucositis (WHO grade ≥3) was significantly more common in *Candida*-positive patients than in non-colonized patients (59.1% versus 23.1%; p = 0.004), highlighting a clinically meaningful interaction between fungal colonization and mucosal injury.

Radiotherapy also profoundly alters the oral microenvironment. Salivary gland damage leads to xerostomia, reduced antimicrobial activity, altered oral pH, and shifts in oral flora, favoring opportunistic fungal proliferation [4]. In the present study, xerostomia was observed in 34/44 (77.3%) *Candida*-positive patients, suggesting a possible association between reduced salivary function and increased fungal colonization during radiotherapy. Mucosal injury and altered oral conditions during treatment may facilitate fungal persistence and colonization. However, the exact nature of this relationship remains multifactorial and cannot be definitively established from the present observational study.

Our findings are comparable to those reported by Rupe et al., who observed an association between oral *Candida* colonization and severe mucositis in patients undergoing radiotherapy for head and neck cancer [11]. Similarly, Chitapanarux et al. documented progressive increases in fungal colonization during radiotherapy [14]. In the present study, oral *Candida* colonization increased during treatment and paralleled increasing mucositis severity; however, these findings should be interpreted as an association rather than evidence of a direct causal relationship. The findings highlight the potential importance of regular oral assessment and supportive care during radiotherapy, particularly in patients with xerostomia or worsening mucosal toxicity.

Comparable findings have been reported in several Indian and regional studies evaluating oral *Candida* colonization among patients undergoing radiotherapy for head and neck cancers. Jain et al. from Gujarat, India, reported a significant increase in oral *Candida* carriage from 20% in healthy controls to nearly 70% among patients with oral cancer receiving radiotherapy and/or chemotherapy, with a predominance of non-albicans *Candida* species [15]. Similarly, Madiyal et al. from Manipal, India, observed a high frequency of oral candidiasis in patients with head and neck malignancy undergoing radiotherapy [16]. They emphasized the role of radiation-induced mucosal injury and xerostomia in promoting fungal overgrowth. Shrestha et al. also demonstrated progressive candidal colonization during different stages of radiotherapy, reporting *Candida* colonization rates reaching up to 93% in irradiated patients with head and neck cancer [17].

The prevalence of *Candida* colonization observed in the present study after the completion of radiotherapy (62.9%) is therefore comparable with previously published Indian data. Similar associations between xerostomia, salivary dysfunction, mucosal barrier disruption, and fungal colonization have also been demonstrated in other regional studies evaluating irradiated patients with head and neck cancer [17,18]. de Freitas et al. reported *Candida* positivity in approximately 58.6% of irradiated patients with head and neck cancer and highlighted the increased prevalence of non-albicans species following radiotherapy [19]. Likewise, Tarapan et al. demonstrated significantly increased *Candida* colonization in xerostomic post-radiotherapy patients, with colonization correlating positively with the severity of dry mouth and reduced salivary flow [18].

Furthermore, several studies have demonstrated that oral *Candida* colonization frequently parallels increasing mucositis severity during radiotherapy [14-21]. Chitapanarux et al. observed fungal colonization in 65.9% of patients during radiotherapy and highlighted the diagnostic overlap between oral candidiasis and radiation-induced mucositis [14]. Rupe et al. similarly demonstrated that *Candida* colonization was associated with severe oral mucositis and may contribute to worsening mucosal toxicity during treatment [11]. The findings of the present study, therefore, support existing evidence that radiotherapy-induced alterations in the oral environment, including xerostomia, epithelial damage, nutritional compromise, and impaired mucosal defense, create favorable conditions for opportunistic fungal colonization and progressive mucosal injury.

Elderly patients in this study were more frequently affected by both candidiasis and severe mucositis. Age-related immune decline, reduced salivary reserve, a higher prevalence of comorbidities such as diabetes and anemia, and diminished oral self-care likely contributed to this increased susceptibility. These factors, combined with radiation-induced mucosal injury, create conditions favorable for fungal colonization and severe mucositis. Nicolatou-Galitis et al. demonstrated that antifungal prophylaxis significantly reduced *Candida* colonization, severe mucositis, and interruptions in radiotherapy [20]. Long-term studies by Grötz et al. further indicate that *Candida* colonization and salivary dysfunction may persist beyond radiotherapy, suggesting the prolonged ecological disturbance of the oral cavity [21].

Although candidiasis in this study was largely transient and resolved in most patients by 12 weeks post-radiotherapy, the high colonization rate during treatment highlights the importance of early detection and preventive strategies. While radiotherapy-induced mucositis cannot be entirely avoided, optimizing oral hygiene, managing xerostomia, controlling systemic comorbidities, and initiating timely antifungal therapy may reduce fungal burden and limit mucosal severity.

Limitations

This study has several limitations. First, the sample size was relatively small and derived from a single tertiary care center, which may limit the generalizability of the findings to broader populations. Second, although microbiological culture and standard identification techniques were performed, detailed species-level characterization, antifungal susceptibility testing, and quantitative fungal load estimation were not systematically evaluated, thereby limiting the assessment of the relationship between fungal burden and the severity of mucosal injury.

Third, several potentially important clinical and treatment-related confounding variables, including nutritional status, baseline oral health, oral hygiene practices, concurrent antibiotic exposure, xerostomia severity, and supportive care adherence, were not analyzed comprehensively and may have influenced both *Candida* colonization and mucositis severity. Tobacco usage history and alcohol consumption were also not evaluated quantitatively as independent variables in the statistical analysis. However, both factors may influence oral mucosal integrity and susceptibility to fungal colonization during radiotherapy.

Additionally, multivariate analysis was not performed; therefore, the independent contribution of individual clinical and treatment-related factors to *Candida* colonization and mucositis severity could not be determined. Treatment compliance, radiotherapy interruptions, and adherence to supportive care measures were also not analyzed separately and may have affected treatment-related outcomes.

Although oral mucositis was assessed using standardized WHO and CTCAE grading systems by trained clinicians, formal interobserver and intraobserver calibration analyses were not performed prior to mucositis grading, which may have introduced observer-related bias and grading variability. Furthermore, the observational design of the study precludes establishing a definitive causal relationship between *Candida* colonization and mucositis severity; therefore, the findings should be interpreted as an association rather than direct causation.

Further multicenter prospective studies with larger sample sizes, detailed microbiological profiling, quantitative fungal assessment, standardized observer calibration, and comprehensive multivariate analysis are warranted to elucidate better the complex relationship between oral *Candida* colonization and radiation-induced oral mucositis in patients with head and neck cancer.

## Conclusions

In conclusion, this prospective observational study demonstrated a progressive increase in oral *Candida* colonization during and after radiotherapy in patients with head and neck cancer, with a statistically significant association between fungal colonization and the severity of oral mucositis. However, given the observational design, this relationship should be interpreted as an association rather than a definitive causal effect. The development and severity of oral mucositis and *Candida* colonization are multifactorial. Several clinical and treatment-related factors, including nutritional status, oral hygiene, comorbid conditions, and supportive care measures, may influence them.

Radiotherapy remains a cornerstone in the management of head and neck cancers, and mucosal toxicity represents a known and manageable adverse effect rather than a limitation of treatment itself. Appropriate pre-radiotherapy oral evaluation, the maintenance of oral hygiene, and timely supportive care are essential in minimizing mucosal complications. The early identification of *Candida* colonization and appropriate management may help reduce symptom burden, improve patient comfort, and enhance treatment adherence. Further well-designed studies with a detailed evaluation of treatment-related variables and confounding factors are required to elucidate better the complex interaction between *Candida* colonization and radiation-induced mucositis.
